# Red cell distribution width and red cell distribution width to total serum calcium ratio as major predictors of severity and mortality in acute pancreatitis

**DOI:** 10.1186/s12876-018-0834-7

**Published:** 2018-07-05

**Authors:** Marta Gravito-Soares, Elisa Gravito-Soares, Dário Gomes, Nuno Almeida, Luís Tomé

**Affiliations:** 1 Department of Gastroenterology, Centro Hospitalar e Universitário de Coimbra, 3000-075 Coimbra, Portugal; 2 Faculty of Medicine, University of Coimbra, 3000-075 Coimbra, Portugal

**Keywords:** Red cell distribution width, Total serum calcium, Acute pancreatitis, Severity, Mortality

## Abstract

**Background:**

Acute pancreatitis (AP) is associated with considerable morbidity and mortality. Current severity scores include multiple variables and some of them are only complete within 48 h of admission. Red cell distribution width (RDW) is a simple and routine parameter that seems to be related to inflammatory status. Our aims were to evaluate the diagnostic value of RDW in severity and mortality of AP comparing with other prognostic scoring systems.

**Methods:**

Retrospective case-control study of a total of 312 patients with AP admitted between 2014 and 2016. Patients with severe AP (cases) were compared with patients with mild AP (controls) in the 1:1 proportion. Additionally, a comparison between survivor and nonsurvivor AP patients was performed. Diagnosis and severity of AP were defined according to the revised Atlanta classification 2012. Variables evaluated included demographics, comorbidities, hospital stay, laboratorial parameters, arterial blood gas analysis, prognostic scores within 24 h of admission (Ranson, BISAP and Modified Marshall) and mortality.

**Results:**

Included 91 cases of severe AP, most males (58.2% vs 51.6%; *p* = 0.228) with mean age of 64.8 ± 16.3 years (vs 67.9 ± 13.7; *p* = 0.239). RDW_0h_ was higher in patients with severe AP (14.6 ± 1.3 vs 12.7 ± 0.5; *p* < 0.001), as well as RDW_0h_-to-serum calcium ratio (1.8 ± 0.3 vs 1.3 ± 0.1; *p* < 0.001). After multivariate and ROC curve analysis, RDW_0h_ (AUROC: 0.960; *p* < 0.001) and RDW_0h_-to-serum calcium ratio (AUROC: 0.973; *p* < 0.001) were the major predictors of severe AP for a cut-off value of 13.0 (S: 92.7%; Sp: 84.3%) and 1.4 (S: 96.3%; Sp: 84.3%), respectively. These factors were superior to prognostic scores, such as Ranson (AUROC: 0.777; *p* < 0.001; cut-off: 3.0), BISAP (AUROC: 0.732; *p* < 0.001; cut-off: 2.0) and Modified Marshall (AUROC: 0.756; *p* < 0.001; cut-off: 1.0). The mortality rate was 8.8% (16/182), all cases associated with severe AP (17.6%; 16/91). RDW_0h_ and RDW_0h_-to-serum calcium ratio were higher in nonsurvivor AP patients (15.3 ± 1.4 vs 13.5 ± 1.3; *p* < 0.001 and 2.0 ± 0.3 vs 1.6 ± 0.3; *p* < 0.001, respectively). In multivariate and ROC curve analysis, RDW_0h_ (AUROC: 0.842; *p* < 0.001; cut-off: 14.0), RDW_24h_ (AUROC: 0.848; *p* < 0.001; cut-off: 13.8) and RDW_0h_-to-serum calcium ratio (AUROC: 0.820; *p* < 0.001; cut-off: 1.7) were independent predictors for AP mortality, superior to conventional prognostic scoring systems Ranson (AUROC: 0.640; *p* = 0.003; cut-off:3.0), BISAP (AUROC: 0.693; *p* = 0.017; cut-off: 2.0) and Modified Marshall (AUROC: 0.806; *p* < 0.001; cut-off:1.0).

**Conclusions:**

RDW is a simple routine parameter, available at admission. This AP cohort showed that RDW_0h_ > 13.0 and RDW_0h_-to-total serum calcium ratio > 1.4 were excellent predictors for severity and RDW_0h_ > 14.0 and RDW_0h_-to-total serum calcium ratio > 1.7 were very-good predictors for mortality, being superior to conventional prognostic scoring systems.

## Background

Acute pancreatitis (AP) is an acute inflammation of the pancreatic parenchyma induced by activated pancreatic enzymes due to multiple causes [1, 2]. Prognosis of AP depends on its severity, currently classified as mild, moderately severe and severe, according to the revised Atlanta classification (RAC) 2012, which emphasizes the presence of persistent organ failure using the modified Marshall (MM) score [3]. Despite most patients have a mild disease, 20% of AP patients develop a severe clinical course associated with significant morbidity and mortality (7–42%) [2]. Early identification of patients at increased risk of severe and fatal AP is crucial to improve prognosis through a prompt medical/endoscopic treatment and admission to a specialized intensive care unit [4]. An ideal marker/prognostic score should be simple, economic, noninvasive, accurate, and quantitative [4, 5]. Several prognostic scoring systems and biological markers have been used to predict severity and mortality in AP [2, 5]. However, most of them are complex and not applicable early enough. Ranson and Bedside Index for Severity in AP (BISAP) are specific and widely used prognostic scoring systems due to easy evaluation and availability in the first 24 h [5]. Single serum markers have also been evaluated, being C-reactive protein (CRP) probably the most useful [4]. However, CRP ≥15 mg/dL at 48 h was found to be a predictor of poor prognosis, and therefore a limited severity discriminator in the early phase of AP [5–7]. Until now, no single serum marker is able to predict severity or mortality in AP at admission. Red cell distribution width (RDW) is a routine parameter of the complete blood count test, described as simple, easy, inexpensive and quantitative that measures the size heterogeneity of peripheral red blood cell (RBC), known as anisocytosis [8]. RDW has been associated with inflammatory markers such as CRP, interleukin-6 and fibrinogen. It revealed to be a good predictor of mortality risk in elderly, critically-ill patients and patients with acute or chronic cardiovascular and respiratory diseases [8–10]. Besides those conditions, RDW has also been evaluated as a mortality predictor in AP [10–13]. However, few recent works showed contradictory results in assessing RDW at admission for AP severity and its relation with well-stablished AP-specific prognostic scores or other serum makers, such as total serum calcium (TSC) [14, 15]. The aims of this study are: (1) to assess RDW ability in predicting AP severity, (2) to assess RDW predictability for AP mortality, and (3) to compare RDW with other serum markers and AP-specific prognostic scoring systems, namely Ranson, BISAP and MM.

## Methods

### Study design and patients

**Fig. 1:**
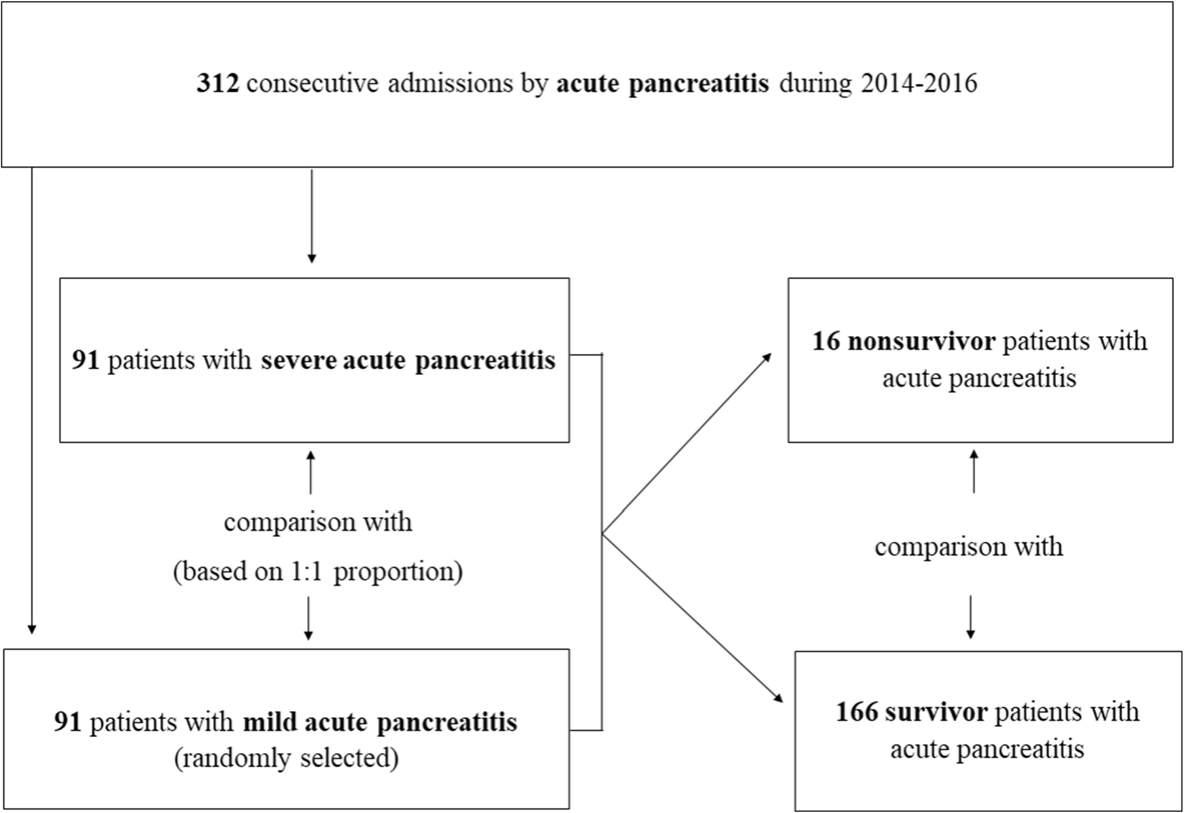
Flow chart of design study

A retrospective case-control study was conducted at the Centro Hospitalar e Universitário de Coimbra from January 2014 to December 2016, including a total of 312 consecutive admissions with AP in our gastroenterology department. Inclusion criteria were age over 18 years-old, a minimum hospital stay of 24 h and a diagnosis of AP according to the RAC [3] (at least two criteria: typical clinical presentation including acute persistent abdominal pain, serum amylase exceeding 3 times the upper limit of normal (100 IU/L) and characteristic findings on abdominal ultrasonography and/or computed tomography). Exclusion criteria included patients with incomplete data and the presence of underlying factors that could change RDW, such as infectious or immunosuppressive conditions/therapy, active malignancy, chronic use of erythropoietin, recent transfusion history, pregnancy or trauma [10, 16]. Ninety-one patients with severe AP were identified during the study period (cases), who were compared with patients with mild AP (controls), in the 1:1 proportion. We randomly selected 91 patients from a total of 146 patients with mild AP to incorporate the control group, using Microsoft Excel (Microsoft, Redmon, WA, USA), and therefore avoid statistical bias. AP severity was also defined according to the RAC in severe, based on the presence of single or multiple persistent organ failure (> 48 h) and/or local complications [3]. Organ failure was classified according to MM including cardiovascular, respiratory and renal failures [3]. In addition, patients who died as a result of AP were compared with AP survivor patients (Fig. 1). Variables evaluated included age, gender, AP etiology, organ failure and local/systemic complications AP-associated, comorbidities, smoking habits (more than 10 cigars/day) and alcohol consumption (more than 20 g alcohol/day). Biochemical and arterial blood gas (ABG) tests at admission were also registered, including white blood cells (WBC) count, aspartate aminotransferase (AST), alanine aminotransferase (ALT), total bilirubin, platelet count, blood urea nitrogen (BUN), creatinine, international normalized ratio (INR), albumin, lactate dehydrogenase (LDH), serum glucose, hemoglobin, hematocrit, serum amylase, C-reactive protein at 0 h (CRP_0h_), CRP at 24 h (CRP_24h_), arterial lactate, arterial partial pressure of oxygen, D-dimers, TSC, RDW at 0 h (RDW_0h_), RDW at 24 h (RDW_24h_). Additionally, RDW_0h_-to-TSC ratio and RDW_0h_-to-platelets ratio were evaluated. RDW was also compared with validated and widely used AP prognostic scores at first 24 h, including Ranson, BISAP and MM scores. The primary endpoints were severity and in-hospital mortality associated with AP.

### Statistical analysis

Statistical analysis was carried out using social package for social sciences version 22.0 for Windows (SPSS Inc., Chicago, IL, USA). The level of significance was set at *p* value less than 0.05. Normality of data distribution was assessed with Kolmogorov-Smirnov or Shapiro-Wilk test. Continuous data were expressed as mean and standard deviation (SD) or median and interquartile range based on the normality of distribution. Groups were compared using Student’s t-test or Mann-Whitney test. Categorical variables were expressed as frequency and percentage and compared using Χ^2^-test or Fisher’s exact test. Multivariate logistic regression analysis with the determination of adjusted odds ratio (aOR) and area under the receiver operating characteristic curve (AUROC) were also applied, assigning the best cut-off in terms of sensitivity and specificity.

## Results

### Characterization of study population

A total of 312 eligible patients were admitted with AP during three consecutive years. One-hundred and forty-six patients had mild AP, 75 (24.0%) moderately severe AP and 91 (29.2%) severe AP. Of the total of 182 enrolled patients (91 with severe AP and 91 with mild AP after random selection), 54.9% (*n* = 100) were males with mean age of 66.3 ± 15.1 years. Most patients (*n* = 102; 56.0%) had gall bladder stones. The most common cause of acalculous AP was alcohol (*n* = 61; 76.2%). An abdominal computed tomography (CT) was performed in 96.7% (*n* = 88) of patients with severe AP. Among these patients, necrotizing pancreatitis was present in 31 patients (34.1%). Twenty-seven (29.7%) patients developed systemic inflammatory response syndrome and 42 (46.2%) patients presented organ failure, the most common being due to respiratory failure (*n* = 30;33.0%) followed by renal failure (*n* = 10;11.0%).

### Determinants of severity in acute pancreatitis

Both groups of patients with severe and mild AP were comparable in terms of age (64.8 ± 16.3 years vs 67.9 ± 13.7 years; *p* = 0.239) and gender (males: 58.2% vs 51.6%; *p* = 0.228), as described in Table 1. After multivariate analysis of significant risk factors in univariate analysis, no significant differences were verified in relation to hospital stay or comorbidities between two groups. Concerning laboratory parameters, there was a significant association of severe AP with high BUN (26.6 ± 17.1 vs 19.4 ± 8.8 years;aOR = 1.002; *p* < 0.004), serum glucose (168.0 ± 72.6 vs 130.9 ± 35.5; aOR = 1.002; *p* < 0.001), RDW_0h_ (14.6 ± 1.3 vs 12.7 ± 0.5; aOR = 1.129; *p* < 0.001), RDW_24h_ (14.3 ± 1.9 vs 12.8 ± 0.5; aOR = 1.015; *p* = 0.005) and RDW_0h_-to-TSC ratio (1.8 ± 0.4 vs 1.3 ± 0.1; aOR = 1.556; *p* < 0.001). In addition, there was also a significant association of severe AP with conventional prognostic scores, namely Ranson (2.6 ± 1.2 years vs 1.5 ± 0.9; aOR = 1.043; *p* < 0.001), BISAP (1.7 ± 0.9 vs 1.0 ± 0.7; aOR = 1.028; *p* < 0.001) and MM (0.8 ± 0.7 vs 0.0 ± 0.0; aOR = 1.184; *p* < 0.001) scores (Table 1).

**Table 1:** Characterization of population according to acute pancreatitis severity

Variable	Severe acute pancreatitis (*n* = 91)	Mild acute pancreatitis (*n* = 91)	*p*U/*p*M	aOR*, 95 CI in M*
Age (yo), μ ± σ	64.8 ± 16.3	67.9 ± 13.7	0.239/−	–
Male gender, n(%)	53(58.2%)	47(51.6%)	0.228/−	–
Alcoholism, n(%)	26(28.6%)	14(15.4%)	0.032/−	–
Smoking, n(%)	0(0.0%)	5(5.5%)	0.023/−	–
Hospital stay, median(IQR)	1.3(2–83)	8.0(2–25)	0.001/−	–
Comorbidities^a^, n(%)	75(82.4%)	83(91.2%)	0.08/−	–
Hypothyroidism	2(2.2%)	9(9.9%)	0.029/−	–
Cerebral vascular accident	9(9.9%)	0(0.0%)	0.002/−	–
Chronic renal disease	10(11.0%)	3(3.3%)	0.004/−	–
Laboratory and gasometrical parameters				
WBC count (cells/mm^3^), μ ± σ	15,630.0 ± 9030.0	31,165.0 ± 1631.7	0.001/−	–
AST (IU/L), μ ± σ	193.5 ± 164.1	778.9 ± 354.6	0.003/−	–
ALT (IU/L), μ ± σ	249.7 ± 180.3	445.5 ± 252.8	0.129/−	–
Total bilirubin (mg/dL), μ ± σ	2.7 ± 2.6	2.2 ± 1.9	0.363/−	–
Platelet count (cells/mm^3^), μ ± σ	212.2 ± 87.1	208.8 ± 72.8	0.975/−	–
BUN (mg/dL), μ ± σ	26.6 ± 17.1	19.4 ± 8.8	0.009/< 0.001	1.002, [1.001;1.007]
Creatinine (mg/dL), μ ± σ	1.5 ± 1.3	0.9 ± 0.3	< 0.001/−	–
INR, μ ± σ	3.0 ± 1.6	1.4 ± 0.2	< 0.001/−	–
Albumin (g/dL), μ ± σ	3.6 ± 0.6	4.0 ± 0.7	< 0.001/−	–
LDH (IU/L), μ ± σ	503.0 ± 403.7	406.8 ± 305.4	< 0.001/−	–
Serum glucose (mg/dL), μ ± σ	168.0 ± 72.6	130.9 ± 35.5	< 0.001/< 0.001	1.002, [1.001;1.317]
Hemoglobin (mg/dL), μ ± σ	13.8 ± 2.6	13.5 ± 1.4	0.406/−	–
Hematocrit (%), μ ± σ	41.5 ± 7.0	40.2 ± 4.3	0.285/−	–
Serum amylase (IU/L), μ ± σ	1315.2 ± 1163.3	2049.2 ± 1715.0	0.398/−	–
CRP_0h_ (mg/dL), μ ± σ	10.9 ± 9.2	5.0 ± 3.3	< 0.001/−	–
CRP_24h_ (mg/dL), μ ± σ	21.1 ± 11.6	11.3 ± 5.8	< 0.001/−	–
Lactate (mg/dL), μ ± σ	2.8 ± 1.7	1.5 ± 1.0	< 0.001/−	–
D-dimers (μg/mL), μ ± σ	6.6 ± 6.5	1.5 ± 0.3	0.192/−	–
Total serum calcium (mg/dL), μ ± σ	8.2 ± 1.2	9.6 ± 0.4	< 0.001/−	–
RDW_0h_ (%), μ ± σ	14.6 ± 1.3	12.7 ± 0.5	< 0.001/< 0.001	1.129, [1.065;1.192]
RDW_24h_ (%), μ ± σ	14.3 ± 1.9	12.8 ± 0.5	< 0.001/0.005	1.015, [1.003;1.055]
RDW_0h_-to-total serum calcium ratio, μ ± σ	1.8 ± 0.4	1.3 ± 0.1	< 0.001/< 0.001	1.556, [1.356;1.756]
RDW_0h_-to-platelets ratio, μ ± σ	0.08 ± 0.03	0.07 ± 0.02	0.03/−	–
Prognostic scores				
Ranson score, μ ± σ	2.6 ± 1.2	1.5 ± 0.9	< 0.001/< 0.001	1.043, [1.001;1.088]
BISAP score, μ ± σ	1.7 ± 0.9	1.0 ± 0.7	< 0.001/< 0.001	1.028, [1.016;1.092]
Modified Marshall score, μ ± σ	0.8 ± 0.7	0.0 ± 0.0	< 0.001/< 0.001	1.184, [1.112;1.256]

*ALT* alanine aminotransferase, *AST* aspartate aminotransferase, *aOR* adjusted odds ratio, *BUN* blood urea nitrogen, *CRP*_*0h*_ C-reactive protein at 0 h, *CRP*_*24h*_ C-reactive protein at 24 h, *CI* confidence interval, *INR* international normalized ratio, *IQR* interquartile range, *LDH* lactate dehydrogenase, *M* multivariate analysis, *RDW*_*0h*_ red cell distribution width at 0 h, *RDW*_*24h*_ red cell distribution width at 24 h, *U* univariate analysis, *WBC* white blood cells

^a^ Only presented significant comorbidities in univariate analysis; Comorbidities evaluated: Neurological, cardiovascular, respiratory and gastrointestinal conditions, malignancy and immunosuppression

### Determinants of mortality in acute pancreatitis

The overall mortality rate was 8.8% (16/182), all cases associated with severe AP, corresponding to a mortality rate of 17.6% (16/91) in the severe AP group. As described in Table 2, cases and controls were similar and therefore comparable in terms of age (71.6 ± 14.3 years vs 65.8 ± 15.1 years; *p* = 0.123) and gender (68.8% vs 53.6%; *p* = 0.245). After multivariate analysis, there were no significant differences between survivors and nonsurvivors regarding hospital stay and comorbidities. Nonetheless, nonsurvivors had higher lactate at admission (3.6 ± 1.8 vs 2.0 ± 1.4; aOR = 1.164; *p* = 0.019), RDW_0h_ (15.3 ± 1.4 vs 13.5 ± 1.3; aOR = 1.038; *p* < 0.001), RDW_24h_ (15.1 ± 1.4 vs 13.4 ± 1.6; aOR = 1.006; *p* = 0.005) and RDW_0h_-to-TSC ratio (2.0 ± 0.3 vs 1.6 ± 0.3; aOR = 1.018; *p* < 0.001) than survivors. Concerning usual prognostic scores, Ranson (2.7 ± 1.2 vs 2.0 ± 1.2; aOR = 1.019; *p* < 0.001), BISAP (2.0 ± 1.0 vs 1.3 ± 0.8; aOR = 1.010; *p* < 0.001) and MM (1.2 ± 1.0 vs 0.6 ± 0.3; aOR = 1.109; *p* = 0.020) scores were also significantly higher in nonsurvivors.

**Table 2:** Characterization of population according to acute pancreatitis mortality

Variable	Nonsurvivors (*n* = 16)	Survivors (*n* = 166)	*p*U/*p*M	aOR*, 95%CI in M*
Age (yo), μ ± σ	71.6 ± 14.3	65.8 ± 15.1	0.123/−	–
Male gender, n(%)	11(68.8%)	5(53.6%)	0.245/−	–
Alcoholism, n(%)	4(25.0%)	36(21.7%)	0.760/−	–
Smoking, n(%)	0(0.0%)	16(9.6%)	0.481/−	–
Hospital stay, median(IQR)	6.5(2–62)	11.0(2–83)	0.071/−	–
Comorbidities^a^, n(%)	15(93.7%)	143(86.1%)	0.391/−	–
Laboratory and gasometrical parameters				
WBC count (cells/mm^3^), μ ± σ	16,810.0 ± 13,490.0	1779.1 ± 1214.1	0.465/−	–
AST (IU/L), μ ± σ	144.6 ± 132.4	271.6 ± 198.1	0.416/−	–
ALT (IU/L), μ ± σ	123.2 ± 100.2	227.8 ± 175.4	0.116/−	–
Total bilirubin (mg/dL), μ ± σ	1.8 ± 1.2	2.4 ± 2.3	0.869/−	–
Platelet count (cells/mm^3^), μ ± σ	174.4 ± 67.0	214.0 ± 80.5	0.057/−	–
BUN (mg/dL), μ ± σ	35.3 ± 20.1	21.9 ± 12.5	0.002/−	–
Creatinine (mg/dL), μ ± σ	2.2 ± 1.4	1.1 ± 0.8	< 0.001/−	–
INR, μ ± σ	1.5 ± 1.0	2.1 ± 1.2	0.018/−	–
Albumin (g/dL), μ ± σ	3.5 ± 0.6	3.8 ± 0.7	0.074/−	–
LDH (IU/L), μ ± σ	465.1 ± 287.8	453.9 ± 332.7	0.207/−	–
Serum glucose (mg/dL), μ ± σ	164.1 ± 104.5	147.3 ± 53.2	0.945/−	–
Hemoglobin (mg/dL), μ ± σ	13.7 ± 3.0	13.7 ± 2.0	0.889/−	–
Hematocrit (%), μ ± σ	41.7 ± 7.5	40.7 ± 5.7	0.630/−	–
Serum amylase (IU/L), μ ± σ	1565.1 ± 1273.3	1703.4 ± 1506.4	0.391/−	–
CRP_0h_ (mg/dL), μ ± σ	10.4 ± 9.6	5.9 ± 3.9	0.046/−	–
CRP_24h_ (mg/dL), μ ± σ	21.8 ± 10.4	15.7 ± 10.3	0.028/−	–
Lactate (mg/dL), μ ± σ	3.6 ± 1.8	2.0 ± 1.4	< 0.001/0.019	1.164, [1.003;1.474]
D-dimers (μg/mL), μ ± σ	9.1 ± 6.8	5.9 ± 4.6	0.042/−	–
Total serum calcium (mg/dL), μ ± σ	8.0 ± 1.1	8.9 ± 1.1	0.001/−	–
RDW_0h_ (%), μ ± σ	15.3 ± 1.4	13.5 ± 1.3	< 0.001/< 0.001	1.038, [1.011;1.087]
RDW_24h_ (%), μ ± σ	15.1 ± 1.4	13.4 ± 1.6	< 0.001/0.005	1.006, [1.001;1.040]
RDW_0h_-to-total serum calcium ratio, μ ± σ	2.0 ± 0.3	1.6 ± 0.3	< 0.001/< 0.001	1.018, [1.007;1.135]
RDW_0h_-to-platelets ratio, μ ± σ	0.10 ± 0.04	0.07 ± 0.03	0.015/−	
Prognostic scores				
Ranson score, μ ± σ	2.7 ± 1.2	2.0 ± 1.2	0.023/< 0.001	1.019, [1.005;1.170]
BISAP score, μ ± σ	2.0 ± 1.0	1.3 ± 0.8	0.003/< 0.001	1.010, [1.003;1.059]
Modified Marshall score, μ ± σ	1.2 ± 1.0	0.6 ± 0.3	< 0.001/0.020	1.109, [1.049;1.169]

*ALT* alanine aminotransferase, *AST* aspartate aminotransferase, *aOR* adjusted odds ratio, *BUN* blood urea nitrogen, *CRP*_*0h*_ C-reactive protein at 0 h, *CRP*_*24h*_ C-reactive protein at 24 h, *CI* confidence interval, *INR* international normalized ratio, *IQR* interquartile range, *LDH* lactate dehydrogenase, *M* multivariate analysis, *RDW*_*0h*_ red cell distribution width at 0 h, *RDW*_*24h*_ red cell distribution width at 24 h, *U* univariate analysis, *WBC* white blood cells

^a^ Only presented significant comorbidities in univariate analysis; Comorbidities evaluated: Neurological, cardiovascular, respiratory and gastrointestinal conditions, malignancy and immunosuppression

### Discriminating performance of RDW and other validated prognostic scores for severity and mortality in acute pancreatitis

**Fig. 2:**
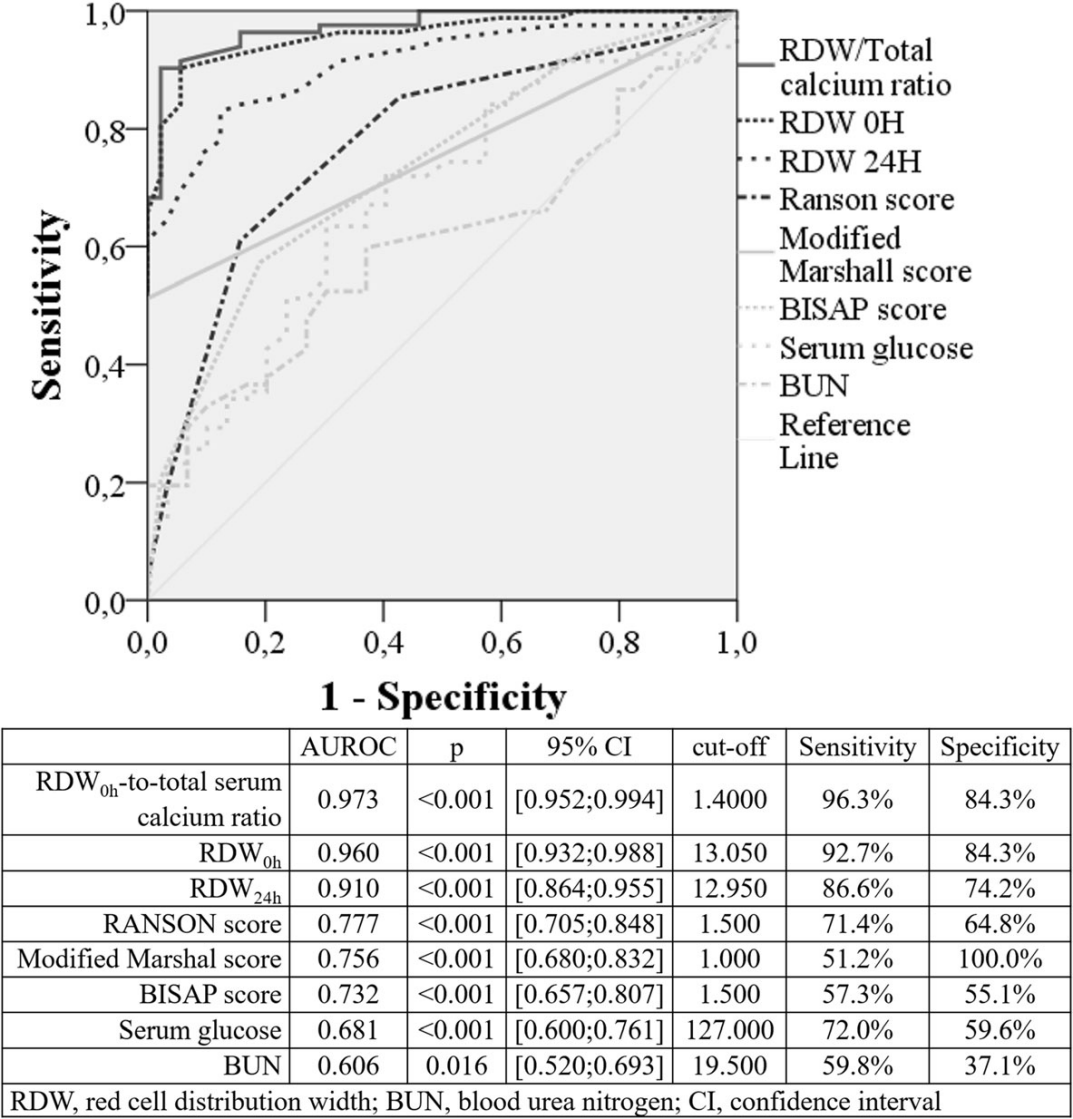
Area under the receiver operating characteristic curve (AUROC) of prognostic scores and independent risk factors for acute pancreatitis severity

In order to assess the discriminant performance of RDW in terms of severity and mortality in AP and to establish a comparison with other independent risk factors, an AUROC analysis was performed. RDW_0h_ (AUROC:0.960; *p* < 0.001) and RDW_0h_-to-TSC ratio (AUROC:0.973; *p* < 0.001) were major predictors of severe AP to a cut-off value of 13.0 (S-92.7%; Sp-84.3%) and 1.4 (S-96.3%; Sp-84.3%), respectively. These factors were superior to well-stablished prognostic scores as Ranson (AUROC: 0.777; *p* < 0.001; cut-off:3.0), MM (AUROC: 0.756; *p* < 0.001; cut-off:1.0) and BISAP (AUROC: 0.732; *p* < 0.001; cut-off:2.0) (Fig. 2).

**Fig. 3:**
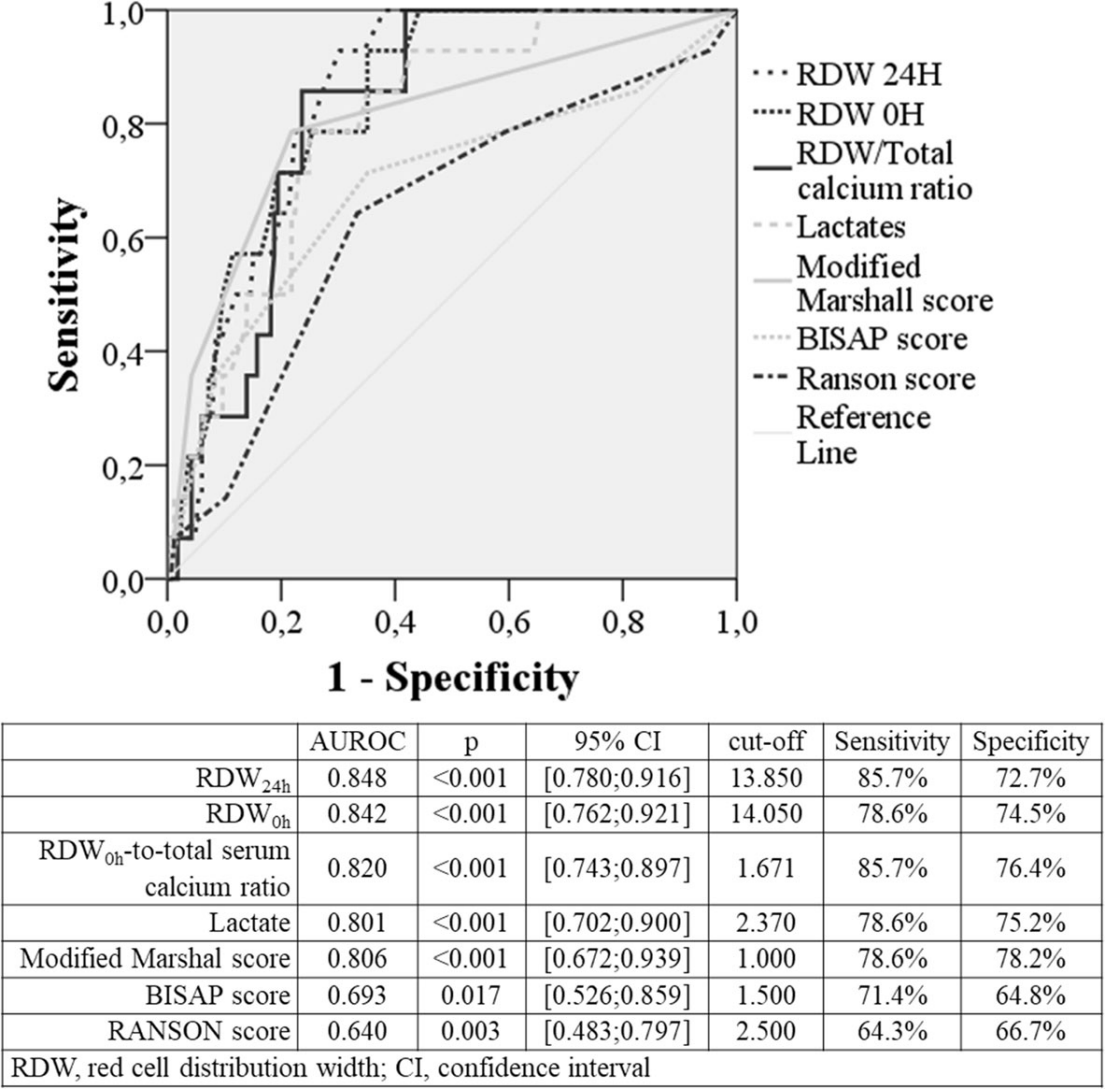
Area under the receiver operating characteristic curve (AUROC) of prognostic scores and independent risk factors for acute pancreatitis mortality

Relatively to mortality in AP and as verified for AP severity, RDW at 0 h and 24 h post-admission were the best predictors for mortality in AP (AUROC: 0.842; *p* < 0.001; cut-off: 14.0 and AUROC: 0.848; *p* < 0.001; cut-off:13.8, respectively), followed by RDW_0h_-to-TSC ratio (AUROC: 0.820; *p* < 0.001; cut-off: 1.7). All of these parameters were better predictors of AP mortality than MM (AUROC: 0.806; *p* < 0.001; cut-off:1.0), BISAP (AUROC: 0.693; *p* = 0.017; cut-off:2.0) or Ranson (AUROC: 0.640; *p* = 0.003; cut-off: 3.0) scores (Fig. 3).

## Discussion

Our study expands the knowledge about early noninvasive predictors of severity and mortality in AP, namely RDW and RDW-to-TSC ratio, since they are simple, inexpensive, quantitative and easy to determine. Furthermore, they proved to have a good-excellent accuracy better than specific-AP prognostic scores, such as Ranson, BISAP and MM, after excluding conditions susceptible to influence RDW and adjusting for confounders.

The assessment of AP severity is crucial to improve prognosis [2, 4, 17]. However, 20–30% of severe AP are misdiagnosed based on clinical data, imaging and biochemical analysis [18]. Over the past decades, several scoring systems have been proposed for early patients’ stratification who have an increased risk of AP morbidity and mortality, with similar accuracy between them [5]. However, no single prognostic score is available for early assessment of AP severity in clinical practice [11]. Two of major scores studied and widely used were Ranson [19] and BISAP [20], since they can be determined in the first 24 h (although Ranson score is only completed within 48 h) and they were specifically developed for AP comparing to others, such as Acute Physiology and Chronic Health Evaluation (APACHE-II) score, which was developed to manage critically ill patients in intensive care units. Accuracy reported for Ranson and BISAP scores were 0.69 and 0.74, respectively [5]. MM score was also recently used to determine AP severity [3]. Other single serum markers of AP severity have been extensively studied, such as hematocrit, creatinine, BUN, TSC, lactate and CRP [4, 7, 21–23]. In addition, some serum pro- and anti-inflammatory markers have been proved to be associated with AP severity, such as interleukin-6, interlekin-8, interleukin-10 and tumor necrosis factor alpha; and serum procalcitonin, a reliable marker of infection/sepsis and useful for predicting infected necrosis in AP. However, the main handicaps of these markers are the fact that they are not routinely used and associated with significant additional costs [2, 6, 22].

The association between arterial lactate and AP mortality was poorly studied [23]. Our study showed that lactate > 2.4 mg/dL (AUROC 0.801) was strongly associated with AP mortality, similar to other work with a reported AUROC of 0.87 [23].

RDW has been considered a remarkable prognostic marker to determine mortality risk in several conditions beyond AP, reflecting inflammation status [8–11]. In a recent systematic review, RDW was independently associated with AP mortality [18]. However, none of published works compared RDW with common prognostic scores [12]. The present study showed that RDW at admission and in the first 24 h were good predictors of AP mortality with AUROC > 0.842, which was higher than in other works with a reported AUROC of 0.66–0.82 [10–12]. For the first time, we also found a strong and independent correlation between RDW_0h_-to-TSC ratio and AP mortality with AUROC of 0.820. In our study, RDW-to-TSC ratio and RDW at admission were superior to conventional prognostic scores in determining AP mortality. Çetinkaya et al. also studied the influence of RDW-to-platelet ratio on AP mortality with an AUROC of 0.783 to a cut-off value of 0.00067 [24]. This marker was also studied in our work, but despite the significance in univariate analysis, it was not an independent prognostic factor after multivariate analysis.

As described in the literature, the present study showed that all AP-associated deaths occurred in patients with severe AP [1, 4], suggesting that finding predictors of AP severity is also crucial in addition to predicting mortality in AP. Despite BUN and serum glucose were associated with AP severity in our study, they did not show a good predictive power (AUROC 0.640 and 0.693, respectively). This association was also verified in other work, with sensitivity of 79% for BUN and 67% for serum glucose [25]. In a recent work, other biochemical parameters were associated with persistent organ failure in AP, including serum albumin with an AUROC slightly higher than Ranson score (0.873 and 0.845, respectively) [26]. However, serum albumin did not reach statistical significance in our study. Regarding RDW, only two studies assessed the relation between this factor and AP severity [14, 15]. A recent work found that RDW could be a useful indicator of AP severity better than serum glucose or TSC (AUROC of 0.801, 0.658 and 0.227, respectively) [15]. However, another recent study concluded that RDW was not a predictor for AP severity, contrarily to BISAP or TSC [14]. In this work, an excellent predictability of AP severity by RDW_0h_ (AUROC 0.960) was verified. For the first time, we also showed a strong and positive association between RDW_0h_-to-TSC ratio and AP severity, representing the best predictor of it (AUROC 0.973). In addition, we verified that conventional AP-specific prognostic scores were reasonable predictors for AP severity (AUROC of 0.777, 0.756 and 0.732 for Ranson, MM and BISAP scores, respectively).

In the past, RDW was widely used for differential diagnosis of anemia. Over the last years, RDW has been associated with systemic inflammation [27] due to the high oxidative stress and inflammatory cytokines that contribute to RDW elevation by reducing RBC survival and maturation, increasing the release of newer and larger RBC into the peripheral circulation and changing of membrane glycoproteins and ion channels of RBC with consequent morphological alteration [10, 12, 28]. Therefore, RDW reflects the degree of inflammation that occurs in AP and thus, can be used to predict its severity. Despite isolated TSC did not represent a good predictor for AP severity in our study, RDW_0h_-to-TSC ratio proved to be an excellent predictor of AP severity and a very good predictor of AP mortality. In fact, hypocalcemia within the first 24 h was associated with AP severity, although its etiopathogenesis is not clearly understood. It has been postulated that hypocalcemia could be related to the calcium soaps formation and parathyroid hormone depletion [21].

There are some limitations, mainly related to the retrospective nature of this study. However, our study groups’ size is within range when compared to other published studies for assessing both severity or mortality in AP [10–12, 14, 15]. Second, RDW samples were collected from a single center and thus, RDW levels could have been slightly different in other populations studied.

## Conclusions

RDW and TSC are simple, inexpensive, noninvasive and quantitative serum markers, provided in a complete blood count test, and therefore readily available on admission. Our study highlights the good predictive power of RDW, evaluated at admission and in the first 24 h, as well as RDW_0h_-to-TSC ratio for both severity and mortality in AP, being superior to Ranson, BISAP and MM scores. RDW_0h_ > 13.0 and RDW_0h_-to-TSC > 1.4 were excellent predictors for AP severity. RDW_0h_ > 14.0 and RD0h-to-TSC > 1.7 were very-good predictors for AP mortality.

Further prospective and multicenter studies are needed to more accurately assess the impact of high RDW as a predictor of severity and mortality in AP and to understand the pathophysiological mechanisms underlying RDW and AP prognosis.

## Abbreviations

ABG: Arterial blood gas
ALT: Alanine aminotransferase
aOR: Adjusted odds ratio
AP: Acute pancreatitis
AST: Aspartate aminotransferase
AUROC: Area under the receiver operating characteristic curve
BISAP: Bedside Index for Severity in Acute Pancreatitis
BUN: Blood urea nitrogen
CRP: C-reactive protein
CRP_0h_: C-reactive protein at 0 h
CRP_24h_: C-reactive protein at 24 h
INR: International normalized ratio
LDH: Lactate dehydrogenase
MM: Modified Marshall score system
RAC: The revised Atlanta classification 2012
RBC: Red blood cell
RDW: Red cell distribution width
RDW_0h_: Red cell distribution width at 0 h
RDW_24h_: Red cell distribution width at 24 h
SD: Standard deviation
TSC: Total serum calcium
WBC: White blood cells
